# Can a Free Wearable Activity Tracker Change Behavior? The Impact of Trackers on Adults in a Physician-Led Wellness Group

**DOI:** 10.2196/resprot.6534

**Published:** 2016-11-30

**Authors:** Lisa Gualtieri, Sandra Rosenbluth, Jeffrey Phillips

**Affiliations:** ^1^ Tufts University School of Medicine Department of Public Health and Community Medicine Boston, MA United States; ^2^ Family Doctors, LLC Swampscott, MA United States

**Keywords:** wearable activity trackers, fitness trackers, trackers, physical activity, chronic disease, behavior change, wellness group, wellness, older adults, digital health

## Abstract

**Background:**

Wearable activity trackers (trackers) are increasingly popular devices used to track step count and other health indicators. Trackers have the potential to benefit those in need of increased physical activity, such as adults who are older and face significant health challenges. These populations are least likely to purchase trackers and most likely to face challenges in using them, yet may derive educational, motivational, and health benefits from their use once these barriers are removed.

**Objective:**

The aim of this pilot research is to investigate the use of trackers by adults with chronic medical conditions who have never used trackers previously. Specifically, we aim to determine (1) if participants would accept and use trackers to increase their physical activity; (2) if there were barriers to use besides cost and training; (3) if trackers would educate participants on their baseline and ongoing activity levels and support behavior change; and (4) if clinical outcomes would show improvements in participants’ health.

**Methods:**

This study was conducted with patients (N=10) in a 12-week physician-led wellness group offered by Family Doctors, LLC. Patients were given trackers in the second week of The Wellness Group and were interviewed 2 to 4 weeks after it ended. The study investigators analyzed the interview notes to extract themes about the participants’ attitudes and behavior changes and collected and analyzed participants’ clinical data, including weight and low-density lipoprotein (LDL) cholesterol over the course of the study.

**Results:**

Over the 12 to 14 weeks of tracker use, improvements were seen in clinical outcomes, attitudes towards the trackers, and physical activity behaviors. Participants lost an average of 0.5 lbs per week (SD 0.4), with a mean total weight loss of 5.97 lbs (*P=*.004). Other short-term clinical outcomes included a 9.2% decrease in LDL levels (*P*=.038). All participants reported an increase in well-being and confidence in their ability to lead more active lives. We identified the following 6 major attitudinal themes from our qualitative analysis of the interview notes: (1) barriers to tracker purchase included cost, perceived value, and choice confusion; (2) attitudes towards the trackers shifted for many, from half of the participants expressing excitement and hope and half expressing hesitation or trepidation, to all participants feeling positive towards their tracker at the time of the interviews; (3) trackers served as educational tools for baseline activity levels; (4) trackers provided concrete feedback on physical activity, which motivated behavior change; (5) tracker use reinforced wellness group activities and goals; and (6) although commitment to tracker use did not waver, external circumstances influenced some participants’ ongoing use.

**Conclusions:**

Our findings suggest that adding trackers to wellness groups comprising primarily older adults with chronic medical conditions can support education and behavior change to be more physically active. The trackers increased participant self-efficacy by providing a tangible, visible reminder of a commitment to increasing activity and immediate feedback on step count and progress towards a daily step goal. While acceptance was high and attitudes ultimately positive, training and support are needed and short-term drop-off in participant use is to be expected. Future research will further consider the potential of trackers in older adults with chronic medical conditions who are unlikely to purchase them, and studies will use larger samples, continue over a longer period of time, and evaluate outcomes independent of a wellness group.

## Introduction

The aim of this pilot research is to investigate the use of wearable activity trackers (trackers) by primarily older adults with chronic medical conditions who have never used trackers previously. This population is among the least likely to purchase trackers and the most likely to face challenges in using them, yet may derive educational, motivational, and health benefits from their use once these barriers are removed. Our study provided free trackers, with training on their setup and use, to patients enrolled in a 12-week physician-led wellness group. Our primary research aims are to determine (1) if participants would accept and use trackers to increase their physical activity; (2) if there were barriers to use besides cost and training; (3) if trackers would educate participants on their baseline and ongoing activity levels and support behavior change in conjunction with the education participants received through the wellness group; and (4) if clinical outcomes would show improvements in participant health.

### Physical Activity

“Regular physical activity is one of the most powerful health promoting practices that physicians and other health care professionals can recommend for patients” [1]. The Physical Activity Guidelines for Americans recommend 150 minutes of moderate-intensity aerobic activity every week, in addition to muscle-strengthening activities at least twice a week, to improve health and lower the risk of chronic conditions [2]. Yet fewer than half of all American adults meet the minimum requirement for physical activity. In some groups, this rate is even lower; only 17.8% of adults aged 45 to 64 and 14.7% of adults aged 65 to 74 meet the aerobic and muscle strengthening physical activity recommendations for their age group, compared to the 25.7% of adults aged 18 to 44 [3].

### Trackers

Trackers are devices that measure health indicators; the most common tracker feature, step count, is set to a default goal of 10,000 steps per day, which is more than the 7000 to 8000 steps per day recommended by the Physical Activity Guidelines [2] and greater than many people can achieve [4]. Tracker sales are growing, with 16.4 million trackers shipped worldwide in the first quarter of 2016 [5]. In the United States, 16% of adults 18 years of age and older who have health insurance have purchased trackers [6]. Almost half of tracker owners are under the age of 35: 42% are 18 to 34 years old, 19% are 35 to 44 years old, 16% are 45 to 54 years old, 16% are 55 to 64 years old, and 7% are 65 and older [7]. Nearly one third of those who buy trackers earn more than US $100,000 a year [8]. Trackers range in price from about US $60 to US $250 for popular brands like Fitbit and Withings, making cost a barrier for many people who may benefit the most from these technologies [9].

Currently, 64% of trackers are purchased for personal use, while 35% of people with trackers received them as a gift or from their employer [10]. In one study, 65% of respondents expressed excitement about their doctors providing trackers [11]. In another study, 48.2% of US adults who do not use a tracker said they would use a free one provided by their physician [12], and 81% of respondents said they would be more likely to monitor health indicators with a device if it was recommended by their health care professional [13].

### Behavior Change

Most people know they should exercise more. In one study, 49.4% of respondents said they tried to increase their exercise in the previous year [14]; however, initiating and maintaining this behavior change is difficult. Self-efficacy, the belief in one’s ability to complete tasks and reach goals, is one of the most consistent predictors of physical activity in adults of all ages, and a lack of self-efficacy is a barrier for many [15]. Other barriers that reduce adherence to behavior change include cost and time [16].

### Use of Trackers to Support Behavior Change

There is a substantial gap between recording information, such as step count, and changing behavior, and limited data exists about the efficacy of trackers in enacting change in physical activity behaviors in any population [17]. Trackers may support behavior change through education and feedback about baseline and ongoing physical activity levels and the ensuing increase in accountability. One study showed that patients dramatically underestimated the number of hours they were sedentary in a day [18], and another found that 69% of tracker users reported that their device improved personal accountability [11].

### Use of Trackers With Older Adults

Several studies have examined the perception and use of trackers in older populations although not in the context of a wellness group setting. One such study found that 58% of 18 to 35 year olds judged trackers as effective, versus 20% of those 56 and older [19]. One study examined the usability and usefulness of trackers as perceived by adults 50 years of age and older with chronic illnesses, and found that trackers helped increase physical activity self-awareness and goal setting [20]. Another study examined how overweight or obese postmenopausal women used trackers and found that, while physical activity levels initially rose, they plateaued after 3 weeks [21]. This study raised the question of how to motivate individuals to achieve further gains after initial success. Finally, another study suggested that new tracker designs and features may be needed for increasing physical activity in people 70 years of age and older [22].

Although those who are older, less affluent, and face significant health challenges are less likely to adopt new technologies, they are more likely to accrue greater benefits from an increase in physical activity, as compared to younger and healthier demographics, once the barriers of cost and training are removed [23]. In one study where adults aged 50 and older tested different trackers for at least 3 days, 73% of the participants felt they would purchase one; however, a lack of instruction manuals and limited familiarity with terminology, such as “link with Bluetooth,” erected barriers to use in this age group [20]. Finally, another study found that adults aged 50 and older were 76% adherent with tracker use in a 6-week pilot study and that 45% reported increased motivation for healthier living, while 46% reported being more active, sleeping better, or eating more healthfully [24]. In addition to removing barriers, increasing physician involvement may also serve to increase tracker effectiveness in changing behaviors. There is an opportunity for health care providers to recommend trackers to patients using data on the risks, benefits, and effectiveness [25], and to assist patients in developing behavior change strategies around tracker use [17].

## Methods

### Setting

Family Doctors, LLC is a private practice in a suburban community north of Boston, MA serving patients of all ages. Two of their physicians, Jeffrey Phillips (JP) and Lisa Ceplikas (LC) cofounded the Family Doctors Wellness Group in 2015 with a nurse practitioner, Wendy Beaumier, and a registered dietitian, Diane Dube. They believed that the “traditional” model of seeing patients in a 15 minute office visit was incompatible with their goal of helping patients develop positive health habits and lifestyle changes. Furthermore, they felt that offering additional wellness coaching in a group setting would take advantage of built-in support from a cohort of patients facing similar challenges. The Wellness Group was designed as a 12-week program with 2 hour meetings every week, during which patients received guidance and teaching from JP and other health experts on physical activity, nutrition, mental health, mindfulness, and sleep.

To be considered for inclusion into the group, patients had to be part of the practice, have at least one chronic medical condition, and be over 18 years of age. Patients were excluded if they didn’t comprehend and speak English or if they had advanced dementia. Recruitment was done through the Family Doctors Facebook page, brochures in the office, and word of mouth. Cost to patients was a US $150 program fee, plus insurance co-payments. Two wellness groups met in 2015, each with an average of 10 participants, and the third started in January 2016. For the third group, which is the focus of this study, recruitment of patients included informal mentions from Family Doctors, LLC staff that patients would receive a free wearable activity tracker. Other than the addition of the tracker, the structure and the demographics of the group were similar to those of the previous wellness groups.

### Participant Demographics

Of the 11 patients who participated in the wellness group from the start, 1 left after eight weeks for personal reasons unrelated to health and is not included in any reported results. For the 10 who completed the program, ages ranged from 39 to 77 years old with a mean age of 61 and a median age of 63. Patients included 2 (20%, 2/10) males and 8 (80%, 8/10) females. Of the 10 primarily lower-income patients, 5 (50%, 5/10) worked full-time, 1 (10%, 1/10) worked part-time, and 4 (40%, 4/10) were retired. All patients suffered from at least one of the following chronic medical problems: overweight and obesity, hypertension, type 2 diabetes, hyperlipidemia, and joint pain. All but 1 of the patients was overweight or obese. Baseline levels of physical activity, as assessed by JP through patient interviews and group counseling, ranged from almost entirely sedentary to moderately active. All patients stated that they were first-time tracker users at the onset of the group.

### Study Structure

At week 2 of the 12-week wellness group, all participants were given a new Withings Pulse, a wearable activity tracker that measures step count, calories burned, distance walked, heart rate, and sleep. Withings Pulse devices were selected for this study because of their availability, to maintain consistency between participants and avoid errors from device variation. Participants were given instructions developed by the research team on the setup and use of the tracker, and JP assisted 7 participants in setting up their devices, while the remaining 3 felt confident in setting up their devices independently. Participants were given guidance on how to select their daily step count goal. Some used the default step goal of 10,000 steps per day, while those with significant physical limitations used a goal personalized to their needs by JP, with instructions to slowly increase their daily and weekly step count as their health permitted.

In alignment with the philosophy of the wellness group, the use of trackers was discussed with participants as a way to build better health habits and create lifestyle change. To generate discussion and provide support, JP used anecdotes from his own tracker use to encourage participants, such as trying to “get in some steps” by taking short walks when possible. In addition, JP helped to troubleshoot or answer participant questions about the trackers during weekly meetings, by phone, and by email.

The study received approval from the Tufts University Health Sciences Institutional Review Board. All 10 who completed the 12-week program consented to participate in semi-structured phone interviews, consisting of 18 open-ended questions with potential follow-up statements to encourage further responsiveness from the interviewees. Lisa Gualtieri (LG) conducted the interviews, which ran for approximately 30 minutes each, and Sandra Rosenbluth (SR) acted as scribe, taking notes to supplement LG’s notes. Interviews took place during a 3-week period to accommodate participants’ schedules. The interviews occurred at weeks 14, 15, and 16; thus participants had used the trackers for 12 to 14 weeks at the time of the interviews. In addition, JP recorded age, systolic blood pressure (SBP), diastolic blood pressure (DBP), low-density lipoprotein (LDL), and body weight at the start and end of the intervention.

### Analysis

Once all interviews were completed, LG and SR independently conducted thematic analysis through reviews of the interview notes to identify underlying themes in participant experiences. Transcripts were manually reviewed for common language and word choice, followed by multiple discussion sessions to determine significance and prevalence of themes. Clinical data recorded by JP were documented in a spreadsheet and analyzed with GraphPad QuickCalcs [26], shown in Table 1. Paired *t* tests and *P* values were calculated, and *P* values less than .05 were considered as significant.

**Table 1:** Changes in systolic blood pressure, diastolic blood pressure, low-density lipoprotein, and body weight after 12 weeks of wearable activity tracker use.

Characteristic	Before	After	Mean change	*P* value
Mean SBP^a^, mmHg	129.8	130.0	0.2	.920
Mean DBP^b^, mmHg	78.0	73.7	–4.3	.113
Mean LDL^c^, mg/dL	105.1	95.4	–9.7	.038
Mean weight, lbs	236.4	230.4	–6.0	.004

^a^SBP: systolic blood pressure.

^b^DBP: diastolic blood pressure.

^c^LDL: low-density lipoprotein.

## Results

### Clinical Outcomes

Participants lost an average of 0.5 lbs per week (SD 0.4), with a mean total weight loss of 5.97 lbs (*P*=.004). Other short-term clinical outcomes included a 9.2% decrease in LDL levels (*P*=.038). Changes in blood pressure were non-significant. These results cannot separate the impact of the wellness group education and support from that of the tracker use.

### Themes

From the interviews following the end of the wellness group, we identified 6 major themes related to the acceptability of, use of, and attitudes towards the trackers by participants.

#### Theme 1: Purchase Barriers

All participants were aware of trackers before receiving one, but none had purchased or used one prior to participation in the wellness group. Half had considered purchasing a device for themselves. For many, the cost of the trackers was an impediment to purchase, but for some the cost was coupled with the lack of perceived value of trackers.

I didn’t want to invest money and then just put it aside. Studies show people fade away from these devices.

I had friends who did it but didn’t seem to get into physical shape, so I thought ‘why bother?’ Also, it was really expensive.

Many participants expressed choice confusion due to the number of brands, models, and features on the market. However, all participants were willing to try a tracker, since being given a tracker removed the barriers of cost, perceived value, and choice confusion.

#### Theme 2: Attitudes Towards the Trackers

When participants were asked to use one word to retrospectively describe how they felt about their tracker when they initially received it, the language varied considerably. Half of the participants, many of whom were individuals who had previously considered purchasing a tracker, expressed excitement and hopeful feelings, such as “grateful” and “thrilled.” In contrast, the other half of the participants expressed negative or neutral feelings, including that they viewed trackers as something for athletes, “a fashion trend,” “a gimmick,” or what “snobby people wore” to maintain “an air of superiority.” Words like “skeptical,” “overwhelmed,” and “unsure” were also used.

Comparatively, when participants described how they felt about their tracker at the time of the interview, all responses were positive. Specific words used include “fantastic,” “helpful,” “optimistic,” “very happy,” “healthy,” “very satisfied,” and “elation.”

A further indication of attitudes towards trackers was that all participants said they would recommend a wearable activity tracker to others. In addition, 8 (80%, 8/10) said they would purchase one as gift for someone else, although 2 (20%, 2/10) expressed concern about the cost. The remaining 2 (20%, 2/10) participants were hesitant about giving a tracker as a gift since they thought it could convey a negative message to the receiver.

#### Theme 3: Trackers as Educational Tools

With initial use, 8 (80%, 8/10) of the participants were surprised by what the tracker reported about their baseline activity level. For example, 40% (4/10 of the participants knew their activity levels were low due to being retired or working in sedentary jobs, but were disappointed to see how low the trackers reported their step counts to be and 2 (20%, 2/10) of the participants reported they were walking more than they thought. In addition, one participant who had low initial numbers expressed that it was important to avoid “doing nothing.”

#### Theme 4: Trackers Provide Feedback on Physical Activity

All participants found it beneficial to have a tangible, visible reminder of a commitment to increasing activity and immediate feedback on step count and progress towards a daily step goal.

...[immediate feedback from the tracker] made me feel like I was making some progress. Before, I would go to the gym and get sweaty, but I didn’t see any changes in the scale or the mirror right away, whereas the tracker was instant gratification.

Another participant described increasing physical activity as something that became “unconscious,” while others mentioned that the tracker served as a trigger to increase their physical activity by adding a walk or increasing the duration of a walk. Some participants expressed frustration that the tracker didn’t register activities such as yoga, standing, or the use of some exercise machines.

Because of the way the machine was, the tracker wasn’t tracking my exercise. I try to use my arms a little when I use that machine.

When describing their tracker use, participants talked about how they felt accountable to it or were kept honest by it.

I don’t have to report to anyone, but I kind of have to report to my tracker. I’m accountable to it in some way.

It’s indisputable. I can’t argue with the numbers that are showing up.

[It] keeps you honest about how much you’re actually exercising.

The tracker has become a part of me.

Participants said they were less likely to make excuses for not being physically active and a few mentioned that they competed with themselves by trying to beat the previous day’s step count, or, if they had a low step count one day, making sure the next day was better. As one said,

It’s a great motivator without making you feel guilty that you didn’t do it.

Participants noted the satisfaction of reaching a goal, exceeding a goal, or setting a new goal.

If I really put my mind to it, it wasn’t that difficult to achieve 7000 or 8000 steps.

It was a real sense of accomplishment to be upping the steps [after increasing the goal from 8000 to 10,000 steps].

[There were] days where I was walking more than expected and I felt good, I wanted to keep that feeling.

#### Theme 5: Tracker Use Reinforced Wellness Group Activities and Goals

Receiving trackers through the wellness group appeared to amplify participants’ positive experiences. Though speculative, participants were asked to consider how they thought their experience would have differed had they used the tracker without the group. Of the participants, 70% (7/10) strongly believed their tracker use would have been less positive without the informal discussion about the tracker and the support from other members during each session. One participant found inspiration from another participant who had a similar work situation, who “helped me figure out how to work on getting out and getting my walks in, especially on days where I felt I couldn’t make the time.” All participants expressed positive sentiments about the wellness group, noting the lack of competition in the group, and that the weekly meetings were “supportive” and provided a sense of “camaraderie.”

#### Theme 6: External Circumstances and Ongoing Use

Participants noted external factors that influenced their tracker use or their ability to incorporate fitness in their lives. Weather was cited by a number of participants, and the constraints of jobs and retirement were also mentioned. Many participants mentioned fluctuations in routine between weekdays and weekends and how the tracker helped them identify and change that.

In the time between receiving the tracker and being interviewed, 40% (4/10) of the participants' use of the trackers or adherence to their fitness routine was temporarily derailed due to external circumstances. One individual stopped walking due to a fall. Another brought the tracker on a trip but didn’t immediately unpack it after returning. A third had trouble fitting exercise into her schedule when a family member was hospitalized. A fourth participant’s tracker stopped working. In all these cases, despite cessation, the intention to continue physical activity remained and was emphasized during the interview process.

Use of the tracker itself varied as well. Only 50% (5/10) of the participants used the wristband with the Withings Pulse. Of the others, 1 (10%, 1/10) alternated between wearing it with the wristband and clipping it to clothing, 2 (20%, 2/10) wore it solely clipped, and 1 (10%, 1/10) kept the device in a pocket. The reasons for solely clipping the device included poor fit and discomfort: in one case due to the circumference of the band and in the other the feel of the band when sweating during menopause. In addition, 1 (10%, 1/10) participant was unable to use the device due to incompatibility with her mobile phone operating system, but used the corresponding Withings app to track step count and thus answered interview questions referring to the impact of tracking on physical activity.

## Discussion

### Principal Findings

Our study findings suggest that adults with chronic medical conditions, when given free trackers in a physician-led group setting, are motivated to increase physical activity behaviors, regardless of their initial attitudes towards trackers. While attitudes were mixed in the beginning, by the end of the study all participants expressed positive attitudes in the interviews, further demonstrated by their commitment to the use of their trackers.

Since, many barriers exist to the purchase and use of trackers, our study was designed to remove cost and training as barriers. We identified perceived value and choice confusion as additional barriers to purchase that our study also removed. We identified ongoing support, in addition to training, as a barrier to use, which was also removed as part of the wellness group. These findings suggest that trackers can have an impact beyond the current consumer base, including with older adults with chronic medical conditions, when support is provided for selecting, purchasing, setting up, and using trackers. Additional potential barriers to use existed on a more individual basis, such as wristband size and material discomfort, which should be considered in larger-scale studies. In this pilot, these barriers were overcome by the tracker brand’s wearability options.

The trackers increased participant self-efficacy by providing a tangible, visible reminder of a commitment to increasing activity and immediate feedback on step count and progress towards a daily step goal (ie, by providing instant gratification when reaching said goal and feedback on the effect of changes to daily routines). Delayed benefits through a lack of feedback or sense of accomplishment can cause physical activity drop-off, a concept known as present-biased preferences. However, the trackers provided feedback that otherwise was only obtainable more slowly through observed changes in clothing sizes or weight on a scale. Having positive emotions associated with physical activity can further increase self-efficacy and is an important motivator for behavior maintenance, which trackers may foster. Identifying barriers and potential methods of increasing self-efficacy in pilot research can support the selection and use of specific theories of behavior change to guide the methodology of future studies. Based on these results from this pilot study, the Transtheoretical Model and Self-Determination Theory may serve as theories that could potentially lead to the development of effective intervention strategies.

Participant’s felt accountable to the trackers, and, when competitive, participants were competing with themselves. The integration of trackers into the wellness group did not foster competitiveness; group discussions focused on sharing tips to increase physical activity and on tracker successes, and lapses were viewed as opportunities for learning. Participants had the same individual goal of creating a healthier lifestyle. The value of this non-competitive atmosphere was emphasized by one participant, who favorably contrasted it to the bullying that took place in a workplace wellness program that incorporated trackers. Trackers may have particular value as impartial aids to increasing physical activity for people who are wary of being judged, are not incented by group competition, or use the tracker feedback to compete with themselves.

In addition to the positive impact that trackers had on participants’ self-efficacy, clinical measures of health improved as well. By the conclusion of the 12-week program, a significant amount of weight was lost. More importantly, the rate of weight loss was consistent with long-term true fat loss [27], thus suggesting that the healthy habits developed over the course of the wellness group were ones that could be maintained over the long-term. In addition, all participants reported an increase in well-being, health education, physical activity, and confidence in their ability to lead more active lives. The success of the wellness group is largely attributable to its multifaceted approach to health and wellness. Nutritional changes, increased physical activity, emotional health, improved sleep, and wellness coaching were all utilized in conjunction with one another to encourage participants to incorporate gradual, evidence-based changes into their lives, promoting true lifestyle change rather than “dieting” or being on an “exercise program” for a limited amount of time. Such wellness group programs can be enhanced by the integration of trackers to serve as reminders during the bulk of the week when not in the wellness group and in the time following the conclusion of the supportive group setting.

### Limitations

Our study possesses several limitations which must be considered in interpreting the results. The most significant limitation was the lack of a control group comprised of an identically structured wellness group that did not provide patients with trackers. As such, some observed results cannot be directly attributed to the trackers. Instead, the results from this small pilot serve to inform the feasibility of research revolving around trackers in physician-guided settings; are trackers accepted and engaged with by participants, when these participants belong to a demographic that does not normally purchase wearable activity trackers? A future study with 3 arms (a wellness group without trackers, an un-enrolled but matched group with trackers, and a wellness group with trackers) would help determine the degree to which certain results can be attributed to certain inputs.

In addition, the small sample size and single study site provide encouraging results but should be replicated and reproduced with more participants and more study sites. Finally, study interviews were conducted between 14 to 16 weeks after the wellness group started and do not provide data on how participants’ motivation to be physically active may change in the longer term.

### Conclusion and Implications

Our findings suggest that adding trackers to wellness groups comprising older participants with chronic medical conditions can increase their self-efficacy and motivation to be more physically active. Barriers need to be identified and removed; in our study, the barriers to purchase included cost, perceived value, and choice confusion were removed by providing participants with free trackers and the barriers to use were removed by providing participants with initial training and ongoing support. Overall, our study demonstrated the educational benefits to individuals of learning their baseline activity levels, the increased self-efficacy arising from concrete feedback on physical activity that motivated behavior change, the positive attitudes that developed towards trackers, and improvements in clinical outcomes.

Other group programs may want to add trackers based on the benefits our study found to adding them in this setting. Furthermore, our findings suggest that it may be cost-effective for physicians and other health care providers to provide free or heavily subsidized trackers, along with training and support, to their patients, especially those who may most benefit from increasing their physical activity. A US $60 activity tracker that lowers the risk of chronic conditions or ameliorates their severity by facilitating changes in health behaviors would be greatly beneficial compared to the health care, medication, or intervention costs required to treat illnesses after they develop. Future research will further consider the potential of trackers in older adults with chronic medical conditions who are unlikely to purchase them, and studies will use larger samples, continue over a longer period of time, and evaluate outcomes independent of a wellness group.

## Abbreviations

DBP: diastolic blood pressure
LDL: low-density lipoprotein
SBP: systolic blood pressure
